# Clinical evaluation of vasomotor system functionality in type 2 diabetic Patients

**DOI:** 10.22088/cjim.8.3.183

**Published:** 2017

**Authors:** Mehdi Maghbooli, Hossein Chiti, Sakineh Taheri, Masoud Asadi-Khiavi

**Affiliations:** 1 Department of Neurology, School of Medicine, Zanjan University of Medical Sciences, Zanjan, Iran.; 2 Zanjan Metabolic Disease Research Center, Zanjan University of Medical Sciences, Zanjan, Iran.; 3 Zanjan Applied Pharmacology Research Center, Zanjan University of Medical Sciences, Zanjan, Iran.

**Keywords:** Diabetes, Vasomotor, Valsalva, Sustained hand-grip, Cold pressor, Mental arithmetic

## Abstract

**Background:**

Autonomic neuropathy and vital organ dysfunctions are the known complications in type 2 diabetes Mellitus (DM). Genetic endowments involving individuals make subtle differences in physiological systems, particularly at the time of sickness. Hence, the presented study was designed to evaluate the vasomotor system in healthy people and type 2 DM cases for determining any functionality differences between the mentioned groups.

**Methods:**

Sixty patients with type 2 diabetes (case group) and sixty healthy subjects (control group) matched for age and sex were enrolled in the study. Then, the performance of vasomotor system was assessed using valsalva maneuver, cold pressor, sustained hand-grip and mental arithmetic tests and the differences were determined via statistical methods.

**Results:**

According to our findings, abnormal response to valsalva maneuver was found in the case group (P=0.028) and the same response was seen about mental arithmetic evaluations. In the case of cold pressor and sustained hand-grip tests, remarkable differences were not found in both groups. Important differences were also found among vasomotor dysfunction and the time of DM labeling.

**Conclusion:**

This study showed a higher incidence of vasomotor dysfunction in DM patients. However, revision in cold pressor and sustained hand-grip tests definition as well as methodology was recommended.

Based on the previous studies, 20% of asymptomatic diabetic patients are involved with some autonomic nervous system dysfunction particularly related to cardiovascular system (1). Diabetic autonomic neuropathy can be involved with one or more organs and manifests following signs and symptoms: Cardiovascular system: chronic sinus tachycardia, lack of exercise heart rate variability, orthostatic hypotension, prolonged QT interval, ischemic heart disease (IHD), silent myocardial infarction (MI) and sudden death. Gastrointestinal tract (GI): dysphasia, gastroparesis, constipation, diarrhea, nausea, vomiting, malabsorption, abdominal pain. Abnormal hidrosis: hyperhidrosis in the upper limb extremities and anhidrosis in the lower limb extremities leading to skin dryness as a risk factor for diabetic foot. Abnormal urinary retention and urinary incontinence happened as well (2, 3). As the most common manifestation of autonomic neuropathy, impotence (erectile dysfunction) occurs in 40% of diabetic men. Neurological complications are associated with duration of diabetes Mellitus (DM) and severity and are usually linked with involvement of other tissues such as retinopathy and nephropathy (4-6). Autonomic neuropathy is a serious and common complication of DM and has not been attentively considered despite its association with increased cardiovascular mortality risk.

Many studies showed that approximately 20% of asymptomatic patients with DM have had impaired autonomic function in cardiovascular system (6-8). Autonomic neuropathy may involve single or multiple organs which affects the nerves that serve the heart, the digestive system, the urinary tract, the sex organs, the sweat glands and eyes. Based on Ewing DJ et al.’s study, the mortality rate in DM patients with impaired autonomic function was 27.5% and increased to 53% during the study period, whereas, the mortality rate during the study period was only 15% as compared to a diabetic patient group with normal autonomic function. Half of the patients with abnormal autonomic function expired due to renal failure and 29% of cases failed because of sudden death. Additionally, poor prognosis was accompanied by autonomic neuropathy, particularly orthostatic hypotension and gastrointestinal abnormalities (9, 10). 

DM autonomic neuropathy, particularly cardiovascular modifications have own values on prognostic implications. On the other hand, the use of multiple evaluating methods in accordance with cardiovascular autonomic neuropathy shows different prognostic values based on sympathetic as well as parasympathetic involvement (11). Treatment of autonomic neuropathy in more advanced stages is very difficult and costly. As a consequence, early diagnosis is important for appropriate treatment and intervention. The vasomotor system evaluation in most of patients have been ignored despite its role in the pathophysiology of diabetes complications. Therefore, early detection of vasomotor disturbances can help to prevent the development of complications as well as resulting in lower morbidity and mortality rates (12-14). Treatment of autonomic neuropathy in the more advanced stages is very difficult and costly and as it has been mentioned, can lead to the patient's sudden death, so early detection is essential for appropriate therapy intervention. Thus, this study was designed to provide some evidence about a series of optimized tests on vasomotor assessments in DM cases (without any neurological problems) which means early detection and effective management of autonomic neuropathy eventually affect the state of health of the patients (15, 16).

## Methods

A total of 60 type 2 DM patients (cases) based on the diagnostic criteria of American Diabetes Association (ADA) standards and 60 healthy subjects (control) were enrolled in this case-control study. Both groups were in the 30-60 years old range. Serial sampling among outpatients was performed to provide the required cases. Exclusion criteria included the risk of other neurological diseases associated with Autonomic Nervous System (ANS) dysfunction, severe heart disease, diabetic nephropathy or other severe nephropathies, and also thyroid disease as well as diseases affecting the nervous system or taking concomitant medications which interfere with the ANS. 

All tools to test and monitor blood pressure and heart rate as well as electrocardiogram examination were organized and launched at the Department of Neurology Vali-e-Asr University Hospital, Zanjan, Iran. The minimum room temperature was 18 °C and the maximum was 28 °C. Average room temperature was kept about 21.4 °C. Vasomotor function was studied in the same condition for all cases. Four following tests were performed on cases through cardiovascular monitoring:1) mental arithmetic test: The subject was asked to subtract 7 from a four digit number (like 1000) for over 4 minutes in a noisy environment and then, the cardiovascular indices (blood pressure and heart rate) were evaluated before and after the test. The cut-off points for normal response is considered as heart rate rising plus systolic pressure increasing over than 5 or 10 mmHg and abnormal response is considered as lack of heart rate rising plus systolic pressure increasing lower than 5 or 10 mmHg. 2) Sustained hand-grip test: Bulb dynamometer machine was used to measure muscle strength. 

The maximum strength of non-dominant hand was measured primarily and then 30% of maximum power was evaluated for 3-5 minutes duration. Throughout the sustained isometric forearm muscle contraction, heart rate and systolic as well as diastolic pressure rise typically (at least 15 mmHg). Two kinds of cut-off points were determined for normal responses; first, normal response is considered as diastolic pressure increasing equal/over than 15 mmHg and borderline response is considered as diastolic pressure increasing equal/over than 11 mmHg but lower than 15 mmHg and eventually, abnormal response is considered as diastolic pressure increasing up to 11 mmHg. In another case, normal response is considered as increasing at least in two parameters and abnormal response is considered as lack of increase at least in two parameters. 3) Valsalva maneuver test: In this test, the subjects were asked to take deep breaths and do an expiratory pressure against the closed glottis for 15 seconds (equal with a force to hold the mercury column at 40 mmHg for 15 seconds) and blood pressure and cardiovascular indices (blood pressure and heart rate) in association with electrocardiography (ECG) were recorded during expiration performance. The ratio of RR interval in the ECG cases obtained through two consequent phases of this maneuver (phases II and IV) was considered for evaluating both groups (cases vs controls). 4) Cold pressor test: The subjects were asked to place their non-dominant hand (up to the wrist) in ice water (4-6 °C) for 1 min and blood pressure changes were measured before and after the test. Cut-off point for normal response is considered as systolic pressure increasing equal/over than 15-20 mmHg associated with diastolic pressure increasing equal/over than 10-15 mmHg and abnormal response is considered as lack of systolic pressure increasing equal/over than 15-20 mmHg in association with diastolic pressure increasing equal/over than 10-15 mmHg (17). The tests were carried out in reasonable intervals to avoid having an effect on each other. All blood pressure measurements were recorded using heart monitors and additionally, the non-dominant arm of all cases were equipped with other barometers to rule out any other confounding factors. Finally, the acquired data were analyzed by statistical tests (independent t-test as well as chi-square test) using SPSS Version 16 software.

## Results

The mean age of the case group was 49.25±6.23 years, the minimum age was 34 years and the maximum age was 60 years. The mean age of the control group was 48.68±6.21 years and their minimum and maximum ages were similar to the cases. The case group included 20 (33.3%) males and 40 (66.7%) females and the control group consisted of 25 (41.7%) males and 35 (58.3%) females. The mean duration of the disease in the case group was 7.95±5.96 years, the minimum and maximum duration of disease were one and 60 years, respectively. The distributions of case quantity according to HbA1c levels were as follows: 13 (21.7%) cases with HbA1c equal/lower than 7%, 16 (26.7%) cases with HbA1c among 7% to 8%, 14 (23.3%) patients with HbA1c lower than 9%, 6 (10%) cases with HbA1c among 9% to 10% and finally 11 (18.3%) patients with HbA1c greater than 10% in the case group. The total number of insulin treated cases was 21 (35%) and the number of patients treated with oral hypoglycemic drugs was 39 (65%). Heart rate and blood pressure increasing more than 5 mmHg was considered as normal response of vasomotor system in mental arithmetic test. According to our findings in mental arithmetic test, 47 (78.3%) patients showed normal whereas in the control group, 56 (93.3%) patients showed normal response (p=0.018). As an alternative analysis of mental arithmetic trial, increase of heart rate and blood pressure greater than 10 mmHg was considered as normal response of vasomotor system to the test. 

Based on the interpretation of this test, 28 (46.7%) patients showed normal response but 32 (53.3%) patients showed abnormal response in the case group. In the control group, 38 (63.3%) patients showed normal response and 22 (36.7%) patients showed abnormal response in this modified version of test and the differences between both groups were not statistically significant (P=0.067). It is similar in the case of sustained hand-grip test with regard to mental arithmetic trial and two methods were used for analyzing and interpreting results based on response thresholds. First, diastolic blood pressure increasing more than 15 mmHg was considered as normal response, between 11 mmHg to 15 mmHg as borderline response and finally, increasing up to 11 mmHg as abnormal response as to sustained hand-grip test. Normal, abnormal and borderline responses to the sustained hand-grip test were found in 34 (56.7%), 18 (30%) and 8 (13.3%) cases, respectively. In the control group, normal, abnormal and borderline responses were shown in 38 (63.3%), 10 (16.7%) and 12 (20%) cases, respectively. The difference between the two groups was not statistically significant (P=0.191). As an alternative method for sustained hand-grip test analysis concomitantly change in at least two parameters namely the heart rate and blood pressure which were considered to have normal response of vasomotor system to the test. Based on this new interpretation of the test, 56 patients of the case group (93.3%) and 59 (98.3%) of control subjects showed normal response (p=0.171). In the case of valsalva maneuver test, Valsalva ratio was obtained based on the patient’s ECG by dividing RR interval in phase IV on the same interval in phase II of valsalva maneuver and then, normal response was subsequently adjusted according to the age of each case. According to the test, normal and abnormal responses were observed in 21 (35%) and 39 (65%) cases, respectively, but in the control group, normal and abnormal responses were observed in 33 (55%) and 27 (45%) cases, respectively. The difference between the two groups was statistically significant (P=0.028). The case was partially repeated in cold pressor test. In the case group, 31 (51%) patients and in the control group, 37 (62.7%) patients showed normal response, (one case was excluded due to inability to test performance) (p=0.22). The results of vasomotor functionality evaluation were provided in table 1.

**Table 1:** Results of vasomotor functionality tests in both case and control groups

**Group** **Variable**	**Control** **N (%)**	**Case** **N (%)**	**P**-_Value_
**Mental arithmetic trial ** #			
Normal response Abnormal response	56 (93.3) 4 (6.7)	47 (78.3) 13 (21.7)	0.018
**Mental arithmetic trial ** ¥			
Normal response Abnormal response	38 (63.3) 22 (36.7)	28 (46.7) 32 (53.3)	0.067
**Sustained hand-grip trial ** †			
Normal response Borderline response Abnormal response	38 (63.3) 12 (20) 10 (16.7)	34 (56.7) 8 (13.3) 18 (30)	0.191
**Sustained hand-grip trial ** ª			
Normal response Abnormal response	59 (98.3) 1 (1.7)	56 (93.3) 4 (6.7)	0.171
**Valsalva maneuver trial**			
Normal response Abnormal response	21 (55) 27 (45)	21 (35) 39 (65)	0.028
**Cold pressor trial ** €			
Normal response Abnormal response	37 (62.7) 22 (37.3)	31 (51.7) 29 (48.3)	0.22

* *P*_*value*_ lower than 0.05 was considered as statistically significant.

# Normal response is considered as heart rate rising plus systolic pressure increasing over than 5 mmHg and abnormal response is considered as lack of heart rate rising plus systolic pressure increasing lower than 5 mmHg

¥ Normal response is considered as heart rate rising plus systolic pressure increasing over than 10 mmHg and abnormal response is considered as lack of heart rate rising plus systolic pressure increasing lower than 10 mmHg

† Normal response is considered as diastolic pressure increasing equal/over than 15 mmHg and borderline response is considered as diastolic pressure increasing equal/over than 11 mmHg but lover than 15 mmHg and finally, abnormal response is considered as diastolic pressure increasing up to 11 mmHg

ª Normal response is considered as increasing at least by two parameters and abnormal response is considered as lack of increasing at least in two parameters

€ Normal response is considered as systolic pressure increasing equal/over than 15-20 mmHg in associated with diastolic pressure increasing equal/over than 10-15 mmHg and abnormal response is considered as lack of systolic pressure increasing equal/over than 15-20 mmHg in associated with diastolic pressure increasing equal/over than 10-15 mmHg

Based on dual cut-off points for mental arithmetic trials using systolic pressure increasing over than 5/10 mmHg, the significance difference was only revealed in the case group when the cut-off points considered as systolic pressure increase over than 10 mmHg (P=0.018). On the other hand, only the patients in case group showed mental arithmetic trial abnormality when we increased our blood pressure range higher than 10 mmHg. The only other abnormal response in the case group was shown in valsalva maneuver (P=0.028). As it is mentioned above, the ratio of RR interval in the ECG cases obtained through two consequent phases of this maneuver (phases II and IV), was considered for evaluating both groups (cases vs control). Other tests did not show any important results. In general, frequency of vasomotor disorder was 65% in case group and 35% in control group without any significant differences (data were not shown).

Cases were also allocated into groups lower or greater than 8% HbA1c as well as lack or existing of vasomotor disorders. HbA1c levels were lower than 8% in 29 cases (23 cases showed vasomotor disorder) and more in 31 cases (13 cases showed vasomotor disorder) and the comparison between these groups did not show statistical differences (P=0.17). As a fact, the three time categories of diabetes are as follows: 1-5 years, 5-10 years and over than 10 years) had major statistical impact on vasomotor disorder in case group (table 2). A total number of 39 cases were treated using oral hypoglycemic agents and 21 cases with insulin injection. The signs and symptoms of vasomotor disorder were found in 71.4% cases of insulin users as well as 61.5% cases of oral hypoglycemic agent users without any statistical significance (P=0.44) between these groups. Multivariate analysis using logistic regression model showed that odds ratio (OR) as an indicator for risk of diabetes was 3.77 times higher those cases with impaired autonomic function. On the other hand, this analysis showed the probability (in their interested CI) and high chance (OR: 3.77) for autonomic function impairment (via considering any autonomic test abnormality as a dependent variable) in DM cases (condition of DM and state of health as independent variables) in comparison with normal cases despite age and gender effects (as another independent variable) which ruled out their multivariate weights using logistic regression method (table 3). Interestingly, this situation was repeated again despite age and gender adjustments (data were not shown).

**Table 2:** The association between tests abnormality and DM duration

**Tests** **Disease Duration**	**Abnormality** € **N (%)**	**Normality ** **N (%)**	**Total (%)**	* ** P**-_Value_
1-5 yr	12 (46.2)	14 (53.8)	26 (100)	0.025
6-10 yr	16 (76.2)	5 (23.8)	21 (100)	
> 10 yr	11 (84.6)	2 (15.4)	13 (100)	
total	39 (65)	21 (35)	60 (100)	

* P-_Value_ lower than 0.05 was considered as statistically significant.

€ Tests abnormality is considered as abnormality in every test which is used for autonomic system evaluation.

**Table 3:** Results according to multivariate analysis using logistic regression model

**Variable**		**Odds ratio (OR)**	**Confidence Interval 95% (CI)**	* ** P**-_Value_
Impaired Autonomic Function	Yes	3.77	1.68–8.46	0.001
	No	1		
Age		0.98	0.92–1.05	0.55
Gender	male	1	0.66–3.13	0.36
	female	1.43		

* P-_Value_ lower than 0.05 was considered as statistically significant.

## Discussion

Based on our findings, demographic properties of the case and control groups did not show significant heterogeneity. Ewing DJ et al. showed that Valsalva maneuver test is valuable in investigating the autonomic system. Fernandez C. et al.’s study showed similar results with our study except in some cases (e.g. sustained hand-grip test results). In our study, Valsalva maneuver test was significantly valuable to investigate the autonomic system (P=0.028) but in sustained hand-grip test the results changed (P=0.171 and 0.191). This mismatching of results in the mentioned studies showed both different types of data analysis (due to different definitions of the normal responses to trials) which were similar to our approach on sustained hand-grip test data (17, 18). 

One of the similar studies which were conducted by Petrofsky J. et al showed that sustained hand-grip test results (especially heart rates) were statistically significant only at the time that case launched about 40% of maximum strength of non-dominant hand. Correspondingly to our findings, this response was not repeated for 10% and 25% of maximum strength in non-dominant hand (19, 20). Sustained hand-grip test was performed again by Petrofsky J. et al for evaluating cases to determine time effect (time prolongation - 5 minutes) on trial results which showed statistically reduction in tolerating following contraction in step II of the test in DM cases in contrast to our findings that showed no statistical significant (data were not shown). The sustained hand-grip and cold pressure tests were performed in the least amount of time and in the step II of sustained hand-grip test, only 4 patients in case group and one case in control group had abnormal responses. Unlike our findings, blood pressure levels at rest and during testing were obviously higher (statistical significant differences) in the case group. Based on the results of this study, impaired vasomotor system is significantly associated with the time of diagnosis of DM. This study showed that vasomotor dysfunction was higher in cases with 6 to 10 years of disease duration, but vasomotor dysfunction rates declined after 10 years of disease partly due to non-uniform distribution of the participant cases (21, 22).Correlation between symptoms and signs of autonomic nervous system failure in DM as observed in other studies which was similar to our findings (23, 24). The study by Dyvberg T. et al. showed that prolongation of DM periods even up to 40 years of morbidity period, affected vasomotor functionality like our findings (25, 26). 

In conclusion a significant relationship between the vasomotor dysfunction as well as prolongation of DM was observed in the present study. Furthermore, it has been shown that the risk of vasomotor dysfunction has no significant relationship with the type of glucose-lowering medications as well as the level of HbA1c. Due to the presented findings, it is recommended that a reevaluation in methodology for cold pressor and sustained hand-grip tests are particularly important for the diabetic cases.

**Ethical issues**

The research followed the tenets of the Declaration of Helsinki; detailed design of the study was explained to the patients and their families and/or legal guardians. The written informed consent was obtained from all subjects and their legal executors. Ethical permission was obtained, all information remained confidential and moral aspects of study were accepted in details of the plan adopted in the Medical Ethics Committee of Zanjan University of Medical Sciences.
